# Gene Therapy for Inherited Retinal Disease: Current Strategies, Personalized Medicine, and Future Implications—A Comprehensive Review

**DOI:** 10.3390/jpm15120619

**Published:** 2025-12-11

**Authors:** Fahad R. Butt, Thanansayan Dhivagaran, Boaz Li, Mark Ashamalla, Brendan K. Tao, Michael Balas, Austin Pereira, Peng Yan, Parnian Arjmand

**Affiliations:** 1 Schulich School of Medicine & Dentistry, University of Western Ontario, London, ON N6A 5B8, Canada; fbutt2027@meds.uwo.ca (F.R.B.);; 2 Faculty of Medicine, The University of British Columbia, Vancouver, BC V6T 1Z4, Canada; bli1025@student.ubc.ca (B.L.); markash@student.ubc.ca (M.A.); 3 Department of Ophthalmology & Vision Sciences, University of Toronto, Toronto, ON M5S 1A1, Canadaaustin.pereira@mail.utoronto.ca (A.P.); pyan@kensingtonhealth.org (P.Y.); 4 School of Medicine, Toronto Metropolitan University, Toronto, ON M5B 2K3, Canada; 5 Mississauga Retina Institute, 1420 Burnhamthorpe Rd. Unit 415, Mississauga, ON L4X 2Z9, Canada

**Keywords:** gene therapy, hereditary retinal diseases, subretinal gene delivery, intravitreal gene therapy

## Abstract

Gene therapy represents a transformative frontier in ophthalmology, offering the potential to address inherited and acquired retinal diseases at their genetic origin rather than through symptomatic management. By introducing exogenous genetic material to restore or modulate gene expression, gene therapy aims to preserve or even restore vision in patients with mutations that disrupt normal retinal function. The eye’s small, compartmentalized structure, relative immune privilege, and direct accessibility through subretinal or intravitreal routes make it an ideal target for localized delivery with minimal systemic exposure. The approval of voretigene neparvovec-rzyl for *RPE65*-mediated retinal dystrophy marked a pivotal milestone, establishing proof of concept for durable and safe gene replacement therapy. Looking ahead, continued refinements in vector design, CRISPR-based editing strategies, and delivery platforms are expected to expand the therapeutic reach of gene therapy beyond monogenic disorders. With multiple early-phase clinical trials underway for inherited and acquired retinal diseases, the coming decade is poised to bring broader applicability, improved durability, and more accessible gene-based treatments across the spectrum of retinal pathology.

## 1. Introduction

### 1.1. Gene Therapy for Eye Disease

Gene therapy represents an innovative method for the treatment of inherited and acquired diseases by introducing exogenous genetic material to alter gene expression for clinical benefit. The primary objective of gene therapy is either to restore function of genes rendered inactivated by mutations, or to mitigate the effects of pathological gene variants [1]. In the context of ophthalmology, gene therapy holds immense potential, as replacing or correcting defective genes prior to significant retinal degeneration can preserve and, in some cases, restore vision [2].

The eye possesses several anatomical and physiological advantages that make it a well-suited target for gene therapy. Its small, compartmentalized structure allows for localized treatment, thereby minimizing systemic exposure and associated risks [2]. Furthermore, the eye’s relative immune privilege helps to reduce immune responses against viral vectors and transgenes, while the blood–retinal barrier limits their unintended systemic spread [3]. The ability to directly access the retina by means of intravitreal or subretinal injections further contributes to the precision and safety of ocular gene therapy [1]. However, recent evidence suggests that ocular immune privilege is not absolute, as some studies have reported inflammatory responses following retinal gene therapy [4].

Several gene therapy strategies have demonstrated clinical success, particularly using adeno-associated virus (AAV) vectors, which have been instrumental in treating inherited retinal disorders (IRDs). The Food and Drug Administration (FDA) approval of Luxturna (voretigene neparvovec-rzyl) in 2017 marked a significant milestone, providing a functional copy of the Retinal Pigment Epithelium-specific 65 kDa protein (*RPE65*) gene to patients with Leber congenital amaurosis type 2 (LCA-2) [5]. Clinical trials of voretigene neparvovec-rzyl demonstrated significant improvements in visual acuity and FST improvements (low light sensitivity) [6]. Despite this success, challenges remain, including the durability of gene therapy effects, vector immunogenicity, and the complexity of addressing diseases caused by multiple genetic mutations [7].

Beyond monogenic diseases, gene therapy is expanding to include polygenic and multifactorial ocular conditions such as age-related macular degeneration (AMD), diabetic retinopathy (DR), and glaucoma. Emerging approaches, such as gene silencing, gene editing, and modifier gene therapy, are being developed to target these complex disorders [8]. Optogenetics, an innovative technique that introduces light-sensitive proteins to retinal cells, is being investigated as a potential therapy for advanced retinal degeneration where traditional gene replacement strategies may no longer be effective [9].

Although the eye is often described as immune-privileged, this protection is incomplete, and viral vectors still interact with local immune pathways. Subretinal delivery exposes vectors to microglia and retinal antigen-presenting cells, while intravitreal injection encounters neutralizing antibodies and complement in the vitreous. These responses can reduce transgene expression, trigger inflammation, such as in gene-therapy–associated uveitis, and limit the feasibility of redosing due to capsid-specific antibody formation. Understanding this balance between immune privilege and vector immunogenicity is essential for ensuring long-term safety and durability of ocular gene therapies.

### 1.2. Personalized Medicine

Personalized medicine, also referred to as precision medicine, represents a novel approach to healthcare that tailors treatments to an individual’s unique genetic, environmental, and lifestyle characteristics. By integrating these variables, precision medicine enables more accurate disease prediction, optimized treatment strategies, and tailored medication regimens. The Precision Medicine Initiative, a national effort launched in the United States, has emphasized the importance of integrating individualized genetic information into clinical practice to improve patient outcomes [10]. Advancements in pharmacokinetics, tissue-specific biomarker discovery, and molecularly targeted therapies have reshaped treatment strategies across various medical fields, including oncology and cardiology [11]. In the field of ophthalmology, personalized medicine is novel and powerful tool for diagnosing and managing eye diseases. Genetic testing has revealed that approximately 40 loci account for 15% to 65% of the variability in AMD pathology [12]. This advancement supports a movement towards early screening and personalized risk stratification, for earlier diagnosis and prevention of irreversible vision loss by earlier intervention [12].

Beyond genetics, personalized medicine in ophthalmology also incorporates artificial intelligence (AI) and pharmacogenomics. AI-supported technologies can assist physicians in analyzing multimodal retinal imaging to detect early signs of DR, AMD and glaucoma, allowing for earlier and more precise interventions [13]. Meanwhile, personalized selection of intraocular pressure (IOP)-lowering medications based on pharmacogenomic variants has shown potential benefits in glaucoma management, mitigating risk of treatment failure and adverse events [14]. These advancements improve the specificity of care, improve patient outcomes and minimize the risk of unnecessary treatment exposure and side effects.

This narrative review explores the current landscape of gene therapy in retinal diseases, highlighting recent advancements, barriers to adoption, and future directions. Although gene therapy has shown substantial promise and early clinical success, its broader implementation remains influenced by practical, economic, and regulatory factors that are discussed in later sections. Additionally, the concept of genetic eligibility raises important questions about equitable access and individualized treatment approaches. By examining the latest developments in gene therapy delivery, the role of genetic testing in treatment selection, and emerging innovations such as Clustered Regularly Interspaced Short Palindromic Repeats (CRISPR)-based editing, this review aims to provide an overview of this evolving field.

## 2. Advances in Retinal Gene Therapy

### 2.1. Overview of Gene Therapy Strategies

#### 2.1.1. Adeno-Associated Virus Vectors

Adeno-associated virus (AVV) vectors are among the most widely used gene delivery systems in ophthalmology due to their ability to transduce quiescent cells, low immunogenicity, and capacity for long-term gene expression in postmitotic tissues [15]. AAV vectors derive from a replication-defective parvovirus and deliver episomal genetic material, reducing the risk of genomic integration-associated mutagenesis [16]. Their primary limitation is a packaging capacity of approximately 5.0 kb, which restricts the size of therapeutic genes that can be delivered [2]. Additionally, AAV-based gene therapies face large scale production challenges [17]. Despite these challenges, AAV-mediated gene therapy has demonstrated remarkable clinical success, exemplified by voretigene neparvovec-rzyl [15]. Ongoing clinical trials continue to expand the applications of AAV vectors to other monogenic retinal disorders.

Beyond these general properties, adeno-associated virus exists as a family of naturally occurring and engineered serotypes with distinct capsid structures and tissue tropism. In the eye, AAV2 has been the most extensively used serotype for subretinal delivery because of its robust transduction of retinal pigment epithelium and photoreceptors, forming the basis for voretigene neparvovec and several other IRD trials. AAV5 and AAV8 also demonstrate strong tropism for outer retinal layers when delivered subretinally and are being explored to optimize transduction efficiency and durability of expression. In contrast, vectors such as AAV2.7m8 and other engineered capsids have been developed to enhance penetration of the inner limiting membrane and improve transduction of inner retinal cells after intravitreal injection, which is particularly relevant for disorders targeting retinal ganglion cells or Müller glia. These serotype-specific differences in tropism and route-of-administration performance are central to vector selection in clinical trial design and will likely shape future indications and delivery strategies for ocular gene therapy (Table 1).

#### 2.1.2. Lentiviral Vectors

Lentiviral (LV) vectors offer an alternative gene delivery system with a larger transgene capacity (~8 kb) and the ability to integrate into the host genome, enabling stable long-term transgene expression [16,18]. LV vectors can transduce both dividing and non-dividing cells, making them suitable for targeting retinal pigment epithelium (RPE) and photoreceptors [18]. While integration into the host genome raises concerns regarding insertional mutagenesis, advances in self-inactivating (SIN) vectors have mitigated these risks [18]. SIN vectors are modified viral vectors in which the enhancer and promoter sequences in the long terminal repeat (LTR) have been deleted, rendering the LTR transcriptionally inactive after integration. This design helps prevent mobilization by replication-competent viruses [19]. LV-based therapies have shown promise in treating retinitis pigmentosa (RP), LCA2, and AMD, particularly given the eye’s compartmentalized nature, which limits systemic dissemination of the vector [1].

#### 2.1.3. CRISPR-Cas9

**Figure 1:**
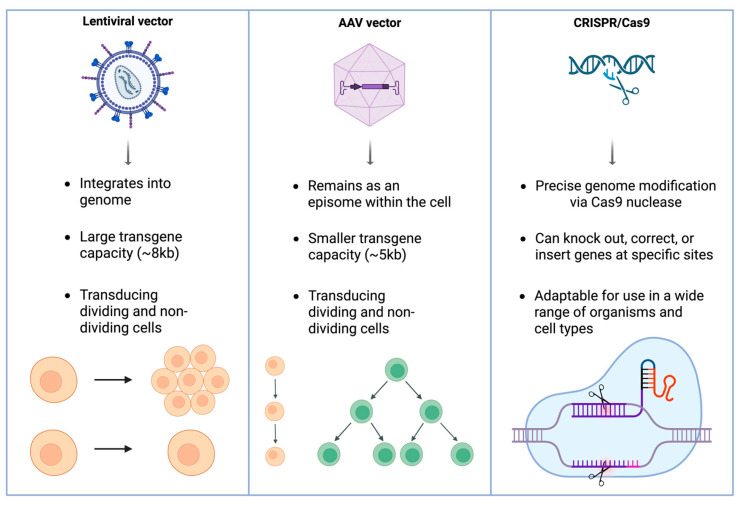
Overview of principal gene therapy platforms in ophthalmology. Lentiviral vectors integrate into the host genome and accommodate large transgenes, AAV vectors remain episomal with smaller cargo capacity, and CRISPR/Cas9 enables precise genome editing for mutation correction or gene knockout. Although AAV vectors can enter both dividing and non-dividing cells, their genomes persist primarily as episomes; thus, in rapidly dividing tissues, episomal dilution reduces long-term expression, whereas post-mitotic retinal cells permit durable transgene expression.

CRISPR-Cas9-based gene editing is a highly precise approach to gene therapy for inherited retinal diseases. CRISPR-Cas9 enables targeted modification of disease-causing mutations, offering the potential to correct the underlying genetic defect, although irreversible retinal degeneration that precedes treatment remains a major limitation [20]. Preclinical studies have demonstrated CRISPR’s efficacy in correcting mutations in a number of different genes [20]. Among these, the CEP290 gene (the most common mutation in LCA) encodes a centrosomal protein found at the connecting cilium of photoreceptors, where it plays a key role in ciliogenesis and the transport of molecules within cilia [21]. In addition, CRISPR has shown promise in targeting the RHO gene, which encodes rhodopsin, a light-sensitive protein in rod cells essential for vision in low light. Mutations in RHO are a common cause of RP [22]. The VEGFA gene, which encodes the vascular endothelial growth factor A protein, is another target of interest due to its key role in stimulating blood vessel formation and its implication in AMD [23]. Despite its promise, CRISPR faces challenges related to delivery efficiency, off-target effects, and immune responses [16]. Ongoing research into base editing, prime editing, and improved delivery vectors aims to enhance CRISPR’s therapeutic potential for ophthalmic applications (Figure 1).

### 2.2. Key Clinical Trials and Approved Therapies

#### 2.2.1. Voretigene Neparvovec-Rzyl (Luxturna)

Voretigene neparvovec-rzyl (*VN*; *AAV2-hRPE65v2*) is the first ocular gene augmentation therapy approved by the FDA for IRDs caused by biallelic *RPE65* mutations [24]. Approved in 2017 by the FDA and in 2018 in the European Union (EU), VN is an AAV2-based vector delivering a functional copy of the *RPE65* gene to viable retinal cells, thereby restoring visual cycle in affected individuals [24,25]. Clinical evidence supporting the efficacy of VN stems from two Phase 1 trials and a pivotal Phase 3 randomized trial, in which treated patients demonstrated significant improvements in multi-luminance mobility testing and full-field stimulus test (FST), indicating improvements in functional vision and residual visual function, respectively [24,26]. These benefits were accompanied by a favorable safety profile [24,26]. Further validation has come from interim analyses of the PERCEIVE study, the largest real-world registry of VN-treated patients to date [24]. The study confirmed sustained FST improvements consistent with prior trials [24]. However, measures such as best-corrected visual acuity (BCVA) and foveal thickness have remained largely unchanged over the two-year follow-up. The PERCEIVE trial included participants aged 2 to 51 years (with the mean age being 19.5 years) and demonstrated consistent long-term safety and efficacy outcomes across this entire age range [24]. These findings further substantiate the durability and safety profile of VN in real-world clinical settings (Table 2).

#### 2.2.2. X-Linked Retinoschisis

X-linked retinoschisis (XLRS) is an X-linked recessive vitreoretinal dystrophy characterized by macular schisis, caused by mutations in the *RS1* gene, which is responsible for encoding retinoschisin, a protein essential for retinal cell adhesion and structural integrity [27]. Gene therapy strategies have focused on delivering a functional copy of *RS1* via AAV vectors to restore homeostatic protein function and retinal cell architecture. In a Phase I/II dose-escalation trial, Pennesi et al. (2022) evaluated intravitreal administration of *rAAV2tYF-CB-hRS1* [28]. The therapy demonstrated an acceptable overall safety profile, although there were observations of chronic uveitis at higher dose levels not responsive to immunosuppressive therapy in 3/27 (11.1%) patients, and 2/27 (7.4%) patients experienced retinal detachment [28]. No significant improvements in BCVA, visual fields (VFs), or electroretinography (ERG) were observed. Similarly, Cukras et al. (2018) tested AAV8-*RS1* on nine patients in a Phase I/IIa trial [29]. There were reports of mild transient intraocular inflammation in 4/9 (44.4%) patients, which were managed and resolved with oral and topical corticosteroid treatments. One patient exhibited transient macular schisis cavity closure, a favorable structural response, although the recurrence of schisis suggests limited durability rather than an adverse event. Otherwise, treatment was overall well tolerated [29]. Despite these promising early findings, further refinement in vector design and administration is needed to optimize therapeutic outcomes.

#### 2.2.3. Stargardt Disease

Stargardt disease, which is the most common hereditary retinal disease, is most often the result of mutations in the *ABCA4* gene. Other less frequent forms of Stargardt disease are caused by mutations in ELOVL4 and PROM1 genes. Stargardt disease leads to progressive central vision loss due to RPE dysfunction and lipofuscin accumulation [30,31]. The *ABCA4* gene encodes a flippase importer protein that facilitates the removal of vitamin A derivatives from photoreceptor cells, thereby preventing their toxic accumulation in the retinal pigment epithelium (RPE) [32]. Parker et al. (2022) conducted a phase I/IIa trial on 22 participants using an equine infectious anemia virus (EIAV) vector encoding *ABCA4* (*EIAV-ABCA4*) [33]. In this study, subretinal injections were well-tolerated across three dose levels, with one case of chronic ocular hypertension. Although one patient demonstrated reduced macular flecks, 6/22 (27%) participants exhibited increased RPE atrophy on fundus autofluorescence as a result of subretinal injection with *EIAV-ABCA4*. While subretinal administration was generally tolerated, the observation that approximately one-quarter of participants developed increased *RPE* atrophy raises important safety concerns and highlights the need for further refinement of *ABCA4* delivery approaches [33]. Beyond ABCA4-associated disease, certain *PRPH2* variants can produce a Stargardt-like macular dystrophy and are relatively common, reflecting the broader genetic heterogeneity within this phenotype. A major challenge for ABCA4 gene replacement therapy is the large size of the ABCA4 coding sequence, which exceeds the packaging capacity of standard AAV vectors and limits the feasibility of traditional AAV-mediated delivery. To overcome this constraint, several alternative platforms including dual-AAV systems, RNA-based therapies, lentiviral vectors, and emerging non-viral technologies are under active investigation to enable efficient delivery of full-length ABCA4 and expand therapeutic options for patients with Stargardt disease.

#### 2.2.4. Age-Related Macular Degeneration (AMD)

Gene therapies for AMD aim to provide sustained anti-VEGF expression, reducing the burden of frequent intravitreal injections. ADVM-022, developed by Adverum Biotechnologies, utilizes an AAV.7m8 vector to express an aflibercept-like protein [34]. The phase 1 OPTIC trial enrolled 30 participants, with 15 in each of the high- and low-dose cohorts, and demonstrated a reduction in anti-VEGF injection frequency of 99% and 85% in the high- and low-dose groups, respectively [34]. Although BCVA and central subfield thickness (CST) remained stable, anterior segment inflammation, particularly in high-dose groups, required long-term steroid management [35]. Similarly, RGX-314 (REGENXBIO Inc.), delivered using AAV8 via subretinal injection (SRI), encodes a ranibizumab-like monoclonal antibody fragment [35]. Early-phase RGX-314 trials involving 42 participants across 5 cohorts have demonstrated promising efficacy, with cohort 3 showing a mean BCVA gain of +14 letters at 2 years, while cohorts 4 and 5 showed changes of +1 and −1 letters, respectively, at 1.5 years. CST changed by +2 µm in cohort 3 and decreased by 46 µm and 93 µm in cohorts 4 and 5, respectively. Additionally, mean annualized anti-VEGF injection frequency was reduced by 66.7% in cohort 3 at 3 years, and by 58.3% and 81.2% in cohorts 4 and 5, respectively, at 1.5 years. While the therapy was generally well-tolerated, localized retinal pigmentary changes were noted at the injection site for 28/42 (67%) of participants [35]. Both ADVM-022 and RGX-314 continue to be evaluated in ongoing trials to determine their long-term safety and efficacy in AMD management.

### 2.3. Challenges with Delivery Methods: Subretinal vs. Intravitreal

**Figure 2:**
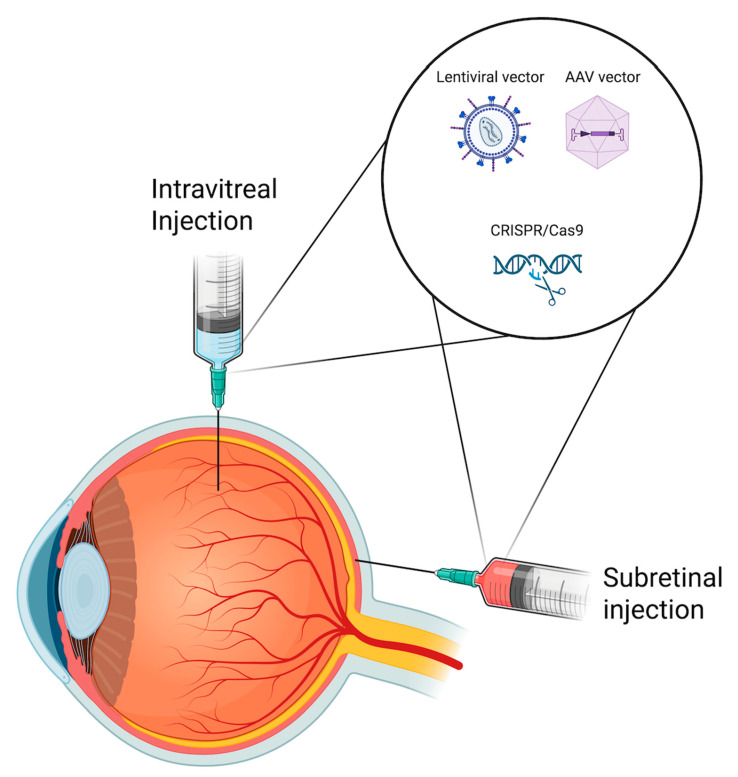
Schematic illustration of subretinal and intravitreal delivery routes used in retinal gene therapy. Gene delivery systems such as adeno-associated viral (AAV) and lentiviral vectors, as well as CRISPR/Cas9-based constructs, can be administered via either approach depending on target cell accessibility and therapeutic objective.

The choice between subretinal and intravitreal administration for gene therapy in ophthalmology presents its own challenges, each with implications on the efficacy and safety of treatment (Figure 2).

SRI allows for direct delivery of viral vectors to the retinal pigment epithelium and photoreceptors, but requires surgical precision to minimize complications [36]. A primary concern is temporary retinal detachment, which can damage photoreceptors and compromise visual acuity, particularly if the fovea is affected, potentially leading to macular hole formation [37]. Additionally, permanent retinal detachment and cataract progression have been observed postoperatively [37]. The procedure also demands meticulous surgical skill, as any subretinal injection inherently creates a localized retinal detachment, and detachment of the fovea is the main concern due to its impact on visual acuity. Reflux of vector material not only reduces efficacy by decreasing the amount delivered to the intended subretinal space, but also increases immune activation as free vector becomes exposed within the vitreous [38]. The mechanical properties of the retina further complicate delivery, as its limited elasticity makes precise vector placement difficult [38]. Moreover, instrument tip tremors (~100 μm) present substantial risks given the narrow margin of the subretinal space [39]. As a result, SRI requires exceptional dexterity.

In contrast, intravitreal injection (IVI) is less invasive and more accessible but has its own limitations. While it allows efficient transduction of inner retinal cells such as ganglion and Müller cells, it is less effective at reaching deeper layers like photoreceptors and the RPE due to the presence of multiple anatomical and physiological barriers [37]. The inner limiting membrane (ILM) serves as a significant diffusion barrier, restricting AAV penetration beyond the inner retina [37]. Additionally, the injected vector faces dilution in the vitreous fluid, reducing its effective concentration before reaching target cells [37,40]. Immune responses further compromise delivery, as neutralizing antibodies in the vitreous can degrade the vector and impair its integrity [40]. Even after internalization by target cells, the vector remains vulnerable to proteasomal degradation, which may prevent successful nuclear transgene delivery [40]. These challenges limit the efficacy of IVI in achieving broad retinal transduction and penetrating deeper layers of the retina.

Looking ahead, several strategies are being explored to overcome the inherent limitations of both subretinal and intravitreal delivery. For SRI, advances in robotic-assisted microsurgery and real-time OCT-guided injection aim to improve precision, reduce foveal trauma, and minimize reflux by stabilizing the needle tip during vector delivery. Novel flexible cannula designs and automated injection platforms may further reduce variability in bleb formation and lower the risk of unintended macular detachment. In parallel, engineered AAV capsids capable of penetrating the inner limiting membrane are driving renewed interest in IVI, offering the possibility of achieving outer retinal transduction without invasive surgery. Techniques such as ILM modulation, suprachoroidal injection, and the development of capsids with enhanced ganglion-cell or photoreceptor tropism are also emerging as promising avenues for improving intravitreal efficiency. Together, these innovations suggest a future where vector design, surgical robotics, and less invasive delivery routes converge to enable safer, more consistent, and broadly applicable gene therapy delivery across a wider range of retinal diseases.

## 3. Personalised Medicine in Gene Therapy

### 3.1. Influence of Genetic Testing on Clinical Outcomes

IRDs occur as a result of mutations in specific genes involved in retinal development, function and maintenance [41]. The genetic complexity of IRDs is reflected in the large number of genetic associations linked to these conditions. To date, nearly 300 genes have been identified whose mutations are linked to phenotypic changes that define specific clinical subtypes [12]. Genetic testing is the sole method by which causative genetic variants can be identified, enabling accurate diagnosis and precise genotype–phenotype correlations [42].

Genotype-phenotype correlation refers to the relationship between specific genetic mutations and their associated clinical manifestations [42]. In gene therapy, understanding genotype-phenotype correlations can help predict disease progression and establish personalized treatment strategies [43]. For instance, pathogenic *BEST1* variants are associated with central visual acuity impairment, leading to Best vitelliform macular dystrophy (BVMD) [42,44]. In contrast, pathogenic variants in *IQCB1* lead to early childhood blindness, which precedes end-stage renal failure in early adulthood seen in Senior-Loken syndrome [45,46]. The phenotypic presentation of a mutation is often based on the degree of functional impairment caused by the altered gene, which can present along a gradient classified as mild (slow-progressing, late-onset, minimal dysfunction) to severe (rapidly progressing, early-onset, and debilitating) [47,48]. Conditions may impact not only the retina but also multiple organ systems and are broadly classified as either syndromic or non-syndromic [47]. For instance, some *USH2A* variants are associated with both retinitis pigmentosa and sensorineural hearing loss, while others cause isolated RP [49,50]. In contrast, *BEST1* variants primarily affect the retinal pigment epithelium (RPE), leading to Best vitelliform macular dystrophy, which typically shows an abnormal electro-oculogram (EOG) without extraocular manifestations [51,52].

### 3.2. Genetic Eligibility

An individual’s genetic mutation, including its location and type, significantly influences the effectiveness and response to prescribed gene therapies. Current gene therapies are designed to address well-characterized, monogenic mutations of specific targetable genes [53]. These therapies depend on accurately identifying the causative gene, as gene replacement targets gene-level dysfunction rather than a specific variant. For example, voretigene neparvovec treats any biallelic pathogenic variant in RPE65, illustrating that gene replacement therapies are directed at the affected gene rather than a single mutation. In contrast, multifactorial conditions like AMD arise from a combination of genetic predisposition and environmental or lifestyle factors [54]. This complexity makes gene therapy options more challenging to develop, as addressing a single genetic factor may not be sufficient to halt disease progression [55,56].

### 3.3. Pharmacogenetics

Gene editing approaches such as CRISPR-Cas9 require molecular interactions within the primary sequence of DNA [57]. Consequently, natural genetic variation amongst individuals can impact treatment efficacy. Variability in each individual’s genome is influenced by parental DNA and somatic mutations that determine their phenotypic traits [58]. Gene therapies target the genome to treat diseases, and genetic variations can thereby influence their effectiveness. For instance, CRISPR-Cas9 gene editing relies on endonuclease cleavage at targeted sites of nucleotide sequences [59]. Cell-based studies have shown that CRISPR-Cas9 editing can result in off-target effects, including unintended alterations at germline single-nucleotide variants (SNVs) [59]. For instance, Yang et al. identified a single-high efficiency off-target site generated by a SNV, and predicted that SNVs have a ~1.5–8.5% of creating off-target sites in human genomes. With numerous CRISPR-Cas9–based retinal gene therapies currently in clinical trials, it is crucial to consider how natural genetic variation across different populations may influence treatment outcomes [60]. Large-scale sequencing datasets now offer valuable insights into human genetic diversity, enabling better assessment of CRISPR endonuclease efficacy across various genomic contexts [61,62,63,64].

Current strategies to enhance specificity include high-fidelity Cas9 nucleases and high specificity vector delivery systems to ensure CRISPR-Cas9 goes to the intended site [65,66]. For instance, in the BRILLIANCE study—a clinical trial targeting CEP290 mutation–associated inherited retinal degeneration—researchers used a high-fidelity Cas9 nuclease, delivered via an AAV5 vector with strong photoreceptor specificity, to administer CRISPR-Cas9 and its associated proteins [67]. Consequently, this minimized the exposure to non-target tissues resulting in no serious adverse events present [67].

## 4. Barriers to Widespread Adoption

### 4.1. Cost and Accessibility

Retinal gene therapies mark a significant advancement in treating retinal diseases. However, their high costs and complex manufacturing processes pose significant challenges to widespread testing and availability. To date, the only approved gene therapy, voretigene neparvovec-rzyl, is priced at USD 425,000 per eye [68,69]. Additionally, as gene therapies are typically one-time treatments, the cost is heavily frontloaded, creating an immediate economic burden for healthcare systems and patients alike [68]. In Canada, the therapy is publicly funded and free for eligible patients, alleviating out-of-pocket expenses. However, this transfers the financial burden to the publicly funded healthcare system, where high upfront costs pose sustainability concerns for provincial budgets.

Economic evaluations suggest that VN provides greater quality-adjusted life-years (QALYs) at a lower overall cost compared to standard medical care without gene therapy, with estimated lifetime costs per patient of $2.2 million versus $2.8 million, respectively [70]. The treatment remains cost-effective if at least 8.8% of its long-term benefits persist beyond the third year post-treatment [70]. However, the main challenge in evaluating gene therapy economic feasibility is the promise of long-term benefits. Long-term outcome data for these therapies remain limited due to their relatively recent introduction. Moreover, because IRDs often manifest from birth, gene therapy candidates are typically young and may be particularly vulnerable to long-term treatment failure or adverse effects. Real-world studies have so far confirmed the safety and effectiveness of these therapies, consistent with clinical trial results. However, with follow-up periods extending only up to two years, these studies are limited in their ability to predict potential long-term complications [24,71].

### 4.2. Ethical and Regulatory Challenges

Ethical concerns surrounding long-term patient outcomes remain a key challenge in the broader adoption of gene therapy. As clinical use is still in its early stages, the absence of long-term data complicates informed consent and patient education. A narrative analysis of patient perspectives revealed a consistent desire for clearer information and transparency, regardless of baseline knowledge [72,73,74,75,76,77]. While many patients reported limited understanding of gene therapy, they generally expressed trust in their healthcare providers’ guidance on its efficacy and safety [72]. However, this reliance suggests that future discoveries of adverse effects may undermine patient-provider relationships and trust.

Emerging gene therapies face many regulatory hurdles during development. First, these therapies are most effective in targeting rare, inherited genetic diseases, making patient recruitment challenging [78]. RP, the most common IRD, affects approximately 1 in 3000–4500 individuals worldwide, while the combined prevalence of all IRDs is around 1 in 3450 [79]. Due to their rarity, clinical trials for IRD gene therapies often face challenges with patient enrollment [80]. To overcome this limitation, ongoing clinical studies involve multi-centre collaborations to increase patient recruitment efforts [81].

### 4.3. Scalability and Infrastructure

Although current gene delivery vectors allow for highly tissue-specific targeting, large-scale vector manufacturing remains a major bottleneck limiting the scalability of gene therapy for clinical trials and widespread use [82]. The transient transfection method is the most widely used system for large-scale AAV production due to its rapid development and turnaround time [82]. Recent studies have reported successful mass production using this approach [83,84]. However, current manufacturing techniques have not yet enabled its widespread use, resulting in production rates that remain insufficient to meet the dosage demands of clinical applications [82]. Additionally, maintaining product yield and quality during rAAV manufacturing poses significant challenges when scaling up from lab bench to mass production [82]. Transient transfection involves incubating a plasmid with growth reagents in cell lines to stimulate rAAV production. Proper mixing and incubation of the transfection reagent with plasmid DNA are essential for maintaining sample homogenization and minimizing variability [82]. While this process is well-controlled on the lab bench using vortexing or shaking, achieving the same level of consistency at a large production scale is challenging [85,86]. Studies have reported productivity losses and intersample inconsistencies, leading to reagent waste and potentially compromising gene therapy efficacy [85,86].

## 5. Future Directions

Recent clinical trials exploring gene therapy use in other ocular regions have emerged, such as achromatopsia and Myocilin-associated Glaucoma (*MYOC*).

Achromatopsia is an autosomal recessive condition characterized by reduced or absent cone function, resulting in severe color blindness, photophobia, nystagmus, and reduced central visual acuity from infancy. It affects 1 in 30,000 individuals and is caused by pathogenic variants that impact cone photoreceptor function [87]. Ongoing clinical trials target specific genetic subtypes and have shown early improvements in color discrimination, with long-term follow-up still underway to assess durability and safety [87].

Glaucoma is a progressive optic neuropathy that often is characterized by increased IOP, either due to increased production or reduced outflow [88]. Mutations in the *MYOC* gene are the most common known cause of inherited glaucoma, affecting an estimated three million people worldwide [89]. These mutations often manifest as juvenile open-angle glaucoma, a form that is typically resistant to conventional pharmacological treatments. Importantly, surgical interventions for glaucoma have a finite duration of effectiveness; procedures performed in early childhood often lose efficacy over time, and repeat surgeries may provide diminishing benefit due to scarring and the progressive loss of viable conjunctival ‘real estate’ needed for aqueous drainage [90,91,92,93]. Given the early onset and invasive nature of standard treatment approaches, gene therapy offers a promising one-time alternative, with strong support from preclinical studies [94]. Researchers successfully developed an animal model that mimics the *MYOC* mutation in humans, demonstrating elevated IOP and glaucomatous progression [95]. Using this model, they demonstrated how CRISPR-Cas9 gene editing can lower IOP in mutated mice and prevent the development of glaucoma [96]. Furthermore, Patil et al. used animal models to demonstrate that LV vectors efficiently deliver Cas9 for gene editing, which gene editing subsequently lowers IOP [97]. Current research provides strong preclinical evidence supporting the efficacy of gene therapy for treating *MYOC*-associated glaucoma. The research group aims to use future studies to focus on minimizing off-target effects of CRISPR-Cas9 base editing, by exploring alternative gene delivery approaches before advancing towards the clinical stage [97].

Current vectors often have adverse effects involving the host’s antiviral defense mechanisms, such as causing intraocular inflammation or loss of transduced, functioning cells after successful treatments [98]. How viral vectors interact with host tissue is predominantly based on non-conserved, surface-exposed proteins and can be modified to preferentially interact with specific host receptors [99]. These sequences can be collected and mapped into a library, with ongoing research using experimental approaches to evaluate their efficacy and specificity [100,101]. However, starting libraries have a plethora of different sequence variations, with many having fundamental issues in genome packaging and assembly. An algorithm to distinguish optimal vectors from all possible sequences will enhance treatment efficacy and reduce off-target effects. AI is an emerging field of research, with specific applications in refining gene delivery vehicles to treat IRDs [102,103]. Recent studies developed AI models having a greater packaging success rate than traditional libraries [95,96,97,98,99,100,101,102,104]. In the future, AI may be able to provide in silico predictions of functional vector candidates over the traditional ‘trial-and-error’ laboratory experiments in validating vector feasibility [105]. This allows a cost-effective and time-efficient approach, by prioritizing high-potential variants before lab testing [98].

**Table 1:** Comparison of Gene Delivery Strategies.

Name	Description	Pros	Cons
Adeno-associated virus (AAV) vectors	Small, non-pathogenic viruses capable of delivering genetic material to both dividing and non-dividing cells. They deliver genetic material as an episome, reducing the risk of insertional mutagenesis [15,16].	Low Immunogenicity [15] Long-term gene expression in post-mitotic or slowly proliferating tissues [15]	Limited carrying capacity (~5.0 kb) [16] Challenges with large-scale production [17]
Lentiviral (LV) vectors	Derived from retroviruses and can infect both dividing and non-dividing cells. They integrate into the host genome, enabling stable, long-term expression [16,18].	Large transgene capacity (~8 kb) [16,18] Low inflammatory response [18]	Risk of oncogenic activation [106] Risk of insertional mutagenesis [18]
Clustered Regularly Interspaced Short Palindromic Repeats (CRISPR)-Cas9	Genome-editing technology that allows for precise DNA modifications, including gene knockout, correction, or insertion [107,108].	High precision and specificity [108]. Can correct mutations at the DNA level [19].	Potential for off-target effects [108]. Ethical and regulatory concerns [109].

**Table 2:** Key Clinical Trials.

First Author	Gene Therapy Mechanism	Treatment Condition(s)	Sample Size (*n*)	Limitations	Success Rate
Russell et al. (2017) [6]	Gene augmentation therapy using AAV2-hRPE65v2 to restore RPE65 function in retinal cells [6].	*RPE65* mutation-associated IRDs, including LCA2 and RP	*n* = 31	No participants under age 4; No data on patients whose visual acuity was better than specified in the protocol (visual acuity 20/60, visual field less than 20 degrees in any meridian) [6].	Mean bilateral MLMT score improved by 1.8 light levels vs. 0.2 in controls at 1 year [6].
Pennesi et al. (2022) [28]	Gene augmentation therapy using intravitreal delivery of *rAAV2tYF-CB-hRS1* to enhance retinal transduction efficiency in XLRS patients [28].	X-linked Retinoschisis (XLRS)	*n* = 27	Ocular inflammation, chronic uveitis, retinal detachments [28,29]	No significant improvements in BCVA, VFs, or ERG [28].
Cukras et al. (2018) [29]	Gene augmentation therapy using intravitreal injection of a self-complementary *AAV8-RS1* vector to restore retinoschisin expression in XLRS patients [29].	X-linked Retinoschisis (XLRS)	*n* = 9	Ocular inflammation, chronic uveitis, retinal detachments [28,29]	BCVA remained within ±10 letters of baseline in all patients over 18 months; no statistically significant ERG changes observed [29]. Transient schisis cavity closure observed in 1 patient (11%); 4/9 patients (44.4%) had dose-dependent ocular inflammation that resolved with treatment [29].
Parker et al. (2022) [33]	Gene augmentation therapy using an equine infectious anemia virus (EIAV) encoding *ABCA4* gene delivered to RPE cells [33]	Stargardt Disease	*n* = 22	RPE atrophy; Ocular hypertension; No clinically significant changes [33]	The treatment was not associated with any clinically meaningful improvements in visual function tests [33]
Busbee et al. (2021) [34]	Gene silencing anti-*VEGF* gene therapy using an AAV.7m8 vector to provide sustained anti-VEGF expression [34].	Age-related macular degeneration	*n* = 30	Ocular inflammation requiring steroid use; Unknown long-term efficacy [34]	93% (high dose) and 67% (low dose) remained injection-free; BCVA was maintained (mean change: −2.5 to +0.2 letters) [34] CRT improved by 19.7 to 132.7 μm across Cohorts 1–3 [34]
Khanani et al. (2022) [35]	Anti-VEGF gene therapy using an AAV8 vector encoding a ranibizumab-like antibody fragment to provide long-term *VEGF* suppression [35].	Age-related macular degeneration	*n* = 42	Postoperative conjunctival hemorrhage; Post operative inflammation; Irritation and pain; Visual acuity reduction [35]	BCVA improved by +14 letters in Cohort 3 at 2 years; Cohorts 4 and 5 had changes of +1 and −1 letters at 1.5 years [35]. 67% showed retinal pigmentary changes; injection burden reduced by 58.3% to 81.2%; CRT change ranged from +2 to −93 µm [35].

AAV = Adeno-associated virus; *ABCA4* = ATP-binding cassette sub-family A member 4; BCVA = Best corrected visual acuity; CRT = Central retinal thickness; EIAV = Equine infectious anemia virus; ERG = electroretinography; IRDs = Inherited retinal diseases; LCA2 = Leber congenital amaurosis type 2; MLMT = Multi-luminance mobility testing; RPE = Retinal pigment epithelium; *RPE65* = Retinal pigment epithelium-specific 65 kDa protein; *RS1* = Retinoschisin 1; RP = Retinitis pigmentosa; *VEGF* = Vascular endothelial growth factor; VFs = Visual Fields; XLRS = X-linked retinoschisis.

## 6. Conclusions

Gene therapy is a promising approach for treating inherited and acquired retinal diseases by targeting underlying genetic etiology rather than symptom management approaches [110]. Although addressing the underlying genetic cause is essential, symptom management remains a critical component of patient care, and patient-reported outcome measures provide valuable insight into functional vision, treatment satisfaction, and real-world quality-of-life impacts. This review has assessed key clinical trials targeting prevalent IRDs, such as X-linked retinoschisis and Stargardt disease, which have all achieved clinical success. However, challenges remain in scalability and vector viability [82,98]. Emerging alternatives, including LV vectors and non-viral delivery systems, offer potential solutions to these challenges but require further optimization to mitigate risks such as insertional mutagenesis and off-target effects. Given the ongoing innovation and advancements in vector engineering, genome editing strategies, and viral system delivery, gene therapy demonstrates the promise of becoming a powerful tool for providing personalized, precision treatment for lifelong IRDs.

## Data Availability

No new data were created or analyzed in this study. Data sharing is not applicable to this article.
